# Ocular phenotype and electroretinogram abnormalities in Lafora disease and correlation with disease stage

**DOI:** 10.1007/s00415-022-10974-7

**Published:** 2022-02-20

**Authors:** Alessandro Orsini, Daniele Ferrari, Antonella Riva, Andrea Santangelo, Angelo Macrì, Elena Freri, Laura Canafoglia, Alfredo D’Aniello, Giancarlo Di Gennaro, Gabriele Massimetti, Carlo Minetti, Federico Zara, Roberto Michelucci, Anupreet Tumber, Ajoy Vincent, Berge Arakel Minassian, Pasquale Striano

**Affiliations:** 1 Paediatric Neurology, Paediatric Department, Santa Chiara’s University Hospital, Azienda Ospedaliero Universitaria Pisana, Pisa, Italy; 2 Ophthalmology Unit, Department of Head/Neck Pathologies, Policlinico San Martino Hospital, IRCCS Hospital-University San Martino, Viale Benedetto XV, 16132 Genoa, Italy; 3 Pediatric Neurology and Muscular Diseases Unit, Department of Neurosciences, Rehabilitation, Ophthalmology, Genetics, Maternal, and Child Health, IRCCS Istituto “G. Gaslini”, Via Gaslini 5, 16148 Genoa, Italy; 4 Department of Neurosciences, Rehabilitation, Ophthalmology, Genetics, Maternal and Child Health, University of Genoa, Genoa, Italy; 5 Department of Pediatric Neuroscience, Fondazione IRCCS Istituto Neurologico “Carlo Besta”, Milan, Italy; 6 Department of Epileptology, Fondazione IRCCS Istituto Neurologico Carlo Besta, Milan, Italy; 7 IRCCS NEUROMED, Pozzilli, Isernia Italy; 8 Section of Psychiatry, Department of Clinical and Experimental Medicine, University of Pisa, Pisa, Italy; 9 Unit of Neurology, IRCCS Istituto delle Scienze Neurologiche di Bologna, Bellaria Hospital, Bologna, Italy; 10 Department of Ophthalmology and Vision Sciences, Hospital for Sick Children, Toronto, ON M5G 1X8 Canada; 11 Division of Neurology, Department of Pediatrics, University of Texas Southwestern Medical Center, Dallas, TX USA

**Keywords:** FFERG, Lafora disease, Neurodegeneration, Progressive myoclonus, Retinal alterations

## Abstract

**Background:**

Lafora disease (LD) is a neurodegenerative disorder featuring action and stimulus-sensitive myoclonus, epilepsy, and cognitive deterioration. Mutations in the *EPM2A/EPM2B* genes classically prove causative for the disease in most cases. Since full-field electroretinogram (ffERG) may reveal early-stage changes in a wide spectrum of diseases, we aimed to evaluate retinal cones and rods dysfunction in a cohort of Italian LD patients.

**Methods:**

Patients with genetically confirmed LD were recruited and subjected to ffERG analysis following the International Society for Clinical Electrophysiology of Vision (ISCEV) protocol.

**Results:**

Six patients aged between 13 and 26 years (mean 19.5 years) were included. The mean age at disease onset was 12.5 years with a mean disease duration of 7 years. The ffERG analysis revealed a global mild to severe generalized cones dysfunction in all patients. Linear correlation was identified between disease stage and the degree of cones and rods dysfunction, as well as between the type of mutation and the cones and rods dysfunction.

**Conclusions:**

This study brings further evidence of early retinal alterations in LD patients. The cones and rods dysfunction grade is related to disease duration. The ffERG is an important tool to determine the disease stage, allowing to evaluate either natural or treatment-related disease progression in a minimally invasive way.

## Introduction

Lafora disease (LD) is a progressive myoclonus epilepsy characterized by the abrupt onset of action and stimulus-sensitive myoclonus in otherwise neurologically normal adolescents [1, 2]. Initial symptoms rapidly turn into progressive dementia, refractory status epilepticus, psychosis, cerebellar ataxia, dysarthria, mutism, and respiratory failure, eventually leading to a severe burden of disability or death within 10 years [1, 3, 4].

LD is primarily caused by mutations of two genes: *EPM2A* and *EPM2B* (*NHLRC1*). Both genes are located on chromosome 6 at q24.3 and p22.3, respectively. The *EPM2A* gene encodes the laforin dual-specificity phosphatase, while the *EPM2B* encodes the malin ubiquitin E3 ligase. These proteins are involved in glycogen metabolism, thus causing the deposition of fibrillary polysaccharides composed of poorly branched glucose polymers, which are called Lafora bodies (LBs). Neuronal LBs mainly localize in dendrites but not in axons, possibly explaining the cortical hyperexcitability reported in LD [4, 5]. A slower disease course with delayed age at death has been reported in most subjects with *EPM2B* mutations [6–8]. Particularly, patients harbouring the p.(D146N) *EPM2B* mutation invariably show atypical milder LD, with delayed disease onset and prolonged disease course [9–11]. Nowadays, next-generation sequencing technologies have shortened the time needed for the diagnosis of several neurological disorders, including LD [12]. Nevertheless, predicting the prognosis and the evolution of LD remains challenging in most patients.

Full-field electroretinogram (ffERG) is a minimally invasive ophthalmological test measuring the electrical activity generated by neural cells in the retina in response to a light stimulus. ffERG can provide diagnostic and prognostic information on a variety of acquired and congenital retinal disorders [13] including retinitis pigmentosa [14], Stargardt disease [15], and Mucopolysaccharidoses [16]. Furthermore, ffERG has been suggested as a useful tool to assess potential retinal toxicity of various treatments [17, 18]. Since recent studies have identified useful ophthalmological biomarkers and displayed early ffERG alterations in LD patients [19], we aimed to evaluate retinal cones and rods dysfunction in a cohort of Italian LD patients.

## Methods

Patients with genetically confirmed LD were recruited for the study. Clinical data including age at disease onset, seizure frequency, and concomitant pharmacological treatments were collected through a standardized questionnaire. The Magaudda Simplified Myoclonus Rate Scaleand a simplified disability scale were used to assess myoclonus severity and walk capability as previously described [9].

For the ophthalmological evaluation, the visual acuity was first measured with the ETDRS visual acuity charts. Red–green color vision was assessed using Ishihara’s test. Then, ffERG was performed following the International Society for Clinical Electrophysiology of Vision (ISCEV) standard protocol, aiming at evaluating the rods and cones electrophysiological responses. After twenty minutes of dark adaptation (DA), patients underwent scotopic ffERG, using a 0.01 cd s/m^2^ flash, which evokes a positive b-wave and represents rod bipolar cells’ activity. The second stimulation was a DA 3.0 cd s/m^2^ flash, eliciting a negative a-wave arising from rod photoreceptors hyperpolarization, which is followed by the positive b-wave reflecting rod bipolar cell depolarization. Then, the DA oscillatory potentials test was performed to evoke responses from amacrine cells, and, after completion, patients were light-adapted (LA) for 10 min through a background luminance of 30 cd/m^2^. Lastly, the cones system was tested using a 3.0 cd s/m^2^ flash stimulus at two different frequencies: 2 Hz photopic ERG and 30 Hz flicker ERG. The 2 Hz frequency aroused an a-wave followed by a b-wave; in this case, the a-wave is driven by cone photoreceptors and cones Off-bipolar cells, whereas the b-wave by cone On- and Off-bipolar cells. The 30 Hz frequency flicker response reflects post-receptoral responses of cones On- and Off-pathways. Finally, spectral-domain optical coherence tomography (SD-OCT) and fundus autofluorescence (FAF) were performed.

### Statistical analysis

We investigated the relationship between disease stage and rods and cones dysfunction by scattergrams and computed Pearson’s correlation coefficients *r/*regression coefficients *b*. Independent sample *t* tests compared the mean level of rods and cones dysfunction observed in the two groups of LD patients (*EPM2A* vs *EPM2B* mutated).

## Results

**Fig. 1:**
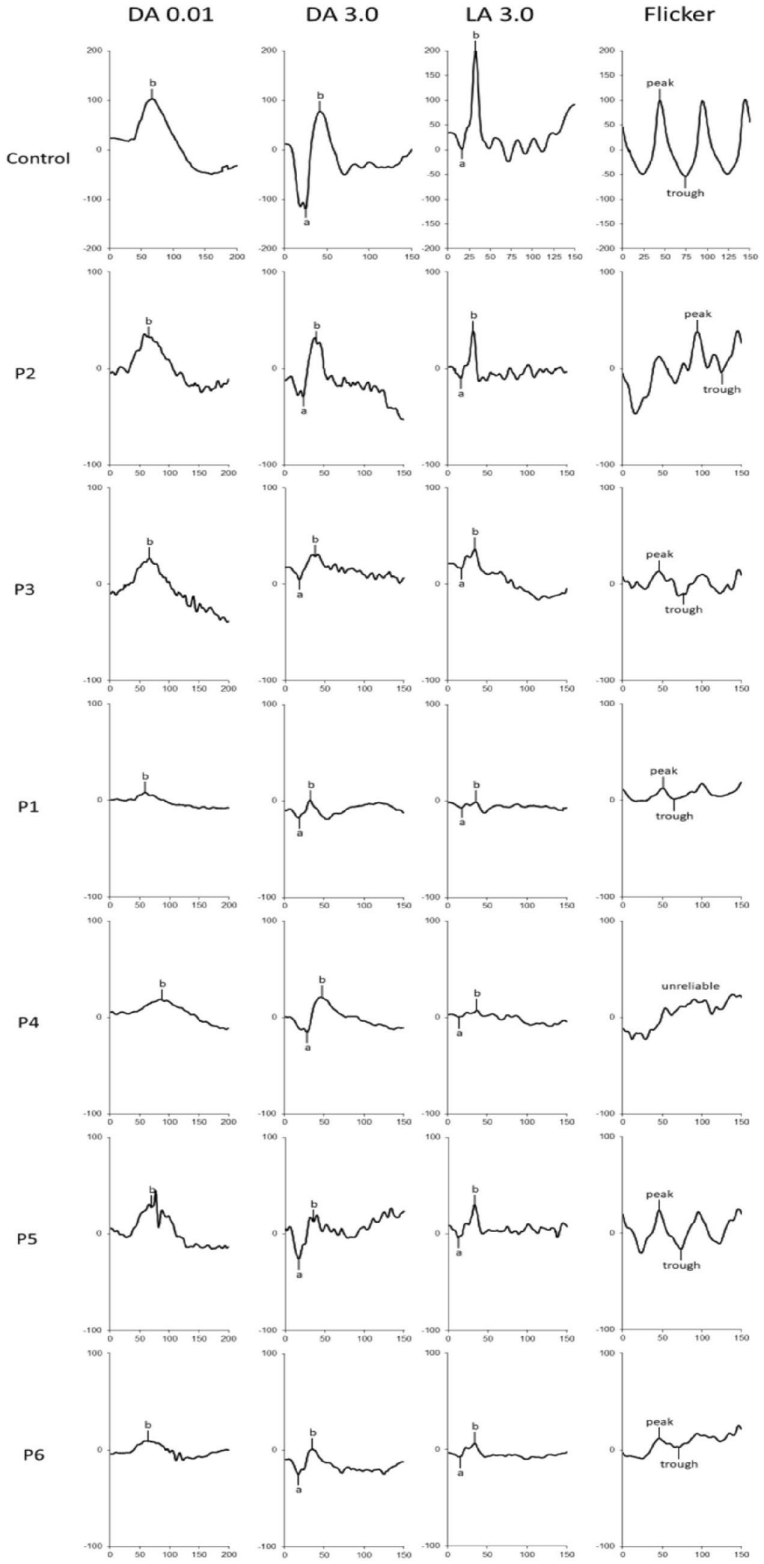
Full-field electroretinogram (ffERG) traces from patients and controls. Dark-adapted (DA) and light-adapted (LA) testing delineate rods and cones system function. Stimulus names include adaptive state of the eye (DA or LA) followed by stimulus intensity in cd.s.m-2. Under DA conditions two different intensity stimulus were used: 0.01, 3.0 cd/s/m^2^. Under LA conditions two different stimuli were used at 3.0 cd/s/m^2^: 2Hz and 30 Hz (Flicker)

**Fig. 2:**
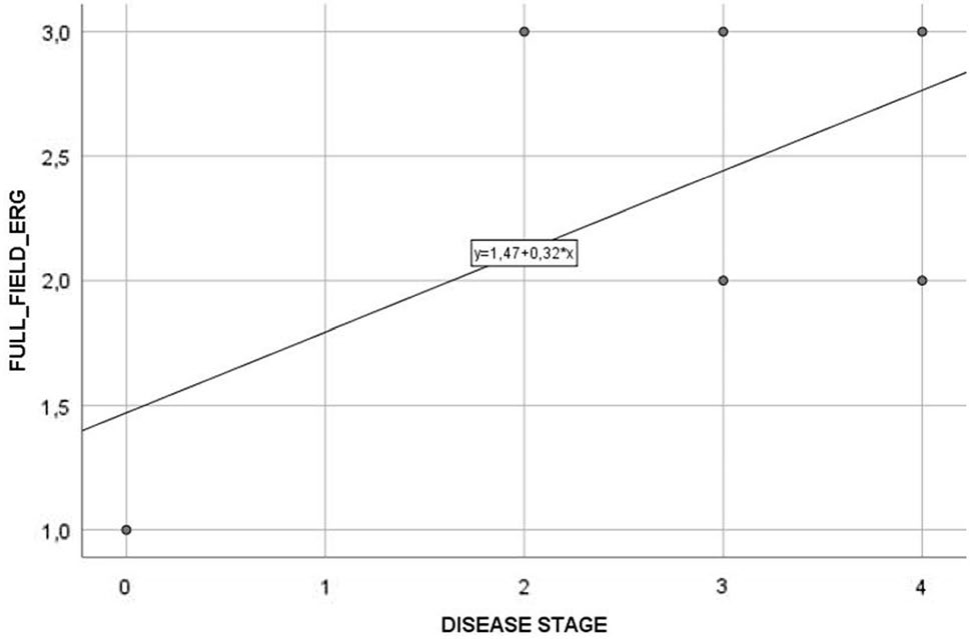
Positive linear correlation observed between LD stage and the grade of cones’ and rodes’ dysfunction at the ERG analysis

**Fig. 3:**
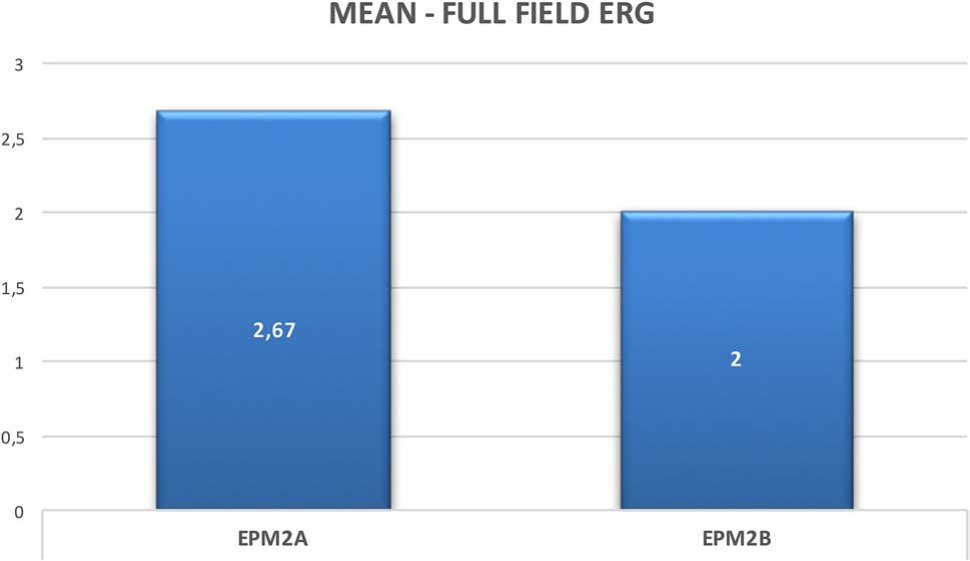
Mean full-field ERG values in the EPM2A and EPM2B mutations subgroups

Six patients (3 *EPM2A*, 3 *EPM2B*) were investigated. Age at evaluation ranged from 13 to 26 years (mean 19.5 years), while age at disease onset ranged from 11 to 16 years (mean 12.5 years) with a mean disease duration of 7 years (range 2–13 years). The myoclonus severity scored between 0 and 4 points (mean, 2.50 points) and the disease stage ranged from 0 to 4 points (mean 2.67 points) (Table 1). The retinal anatomy, FAF, SD-OCT, visual acuity, and color vision tests were unremarkable in all individuals. No patients showed retinitis pigmentosa, excluding retinal pigment epithelial atrophy, or any significant structural abnormality of the photoreceptor outer segments in the central retina (50°). We did not detect structural alterations in the macula on SD-OCT. The ffERG analysis revealed a generalized mild to severe cones dysfunction in all patients, traces are reported in Fig. 1, whereas raw values are displayed in Table 2. Specifically, mild cones dysfunction was detected in one patient (#P2; amplitude deviation RE/LE: − 3.3/− 3.5), whereas a moderate dysfunction was found in patients #P3 and #P5 (mean amplitude deviation RE/LE: − 5.5/− 5.6), and severe cones dysfunction was noted in three patients #P1, #P4, and #P6 (mean amplitude deviation: − 9.2/− 8.6) (Table 1). A positive correlation coefficient (*r* = 0.597) between the LD stage and the degree of cones and rods dysfunction at the ffERG analysis was observed; the linear relationship is well represented by the regression line (*y* = 1.47 + 0.32*x) (Fig. 2). Table 3 shows the Pearson correlation coefficients. For full-field ERG, we found a stronger linear correlation (*r* 0.597, *p* value 0.211) between cones and rods dysfunction and the disease stage. We found a moderate/strong correlation for photopic (PHOT) LE (*r* 0.525, *p* value 0.285) and ERG-LE (*r* 0.407, *p* value 0*.*211), whereas a weak correlation has been observed for scotopic (SCOT) LE (*r* − 0.184, *p* value 0.727). However, *t* test analysis for the EPM2A and EPM2B subgroups displayed no significant difference for ffERG (2.67 ± 0.58 vs. 2.00 ± 1.0, *p* value 0.374), (Fig. 3) ERG-LE (− 5.67 ± 1.22 vs. − 4.17 ± 1.52, *p* value 0.253), PHOT-LE (− 7.37 ± 2.76 vs. − 6.60 ± 3.35, *p* value 0.775) and SCOT-LE (− 7.03 ± 3.04 vs. − 5.40 ± 1.78, *p* value 0.467), as shown in Table 4.

**Table 1:** Demographics, clinical and genetic data, and ffERG results of LD patients

PtID	Gender	Age at last evaluation (years)	Age at onset (years)	Disease duration (years)	ffERG results	Amplitude deviation (SD) RE/LE	Disease stage at the examination	Genetic findings
P1	M	18	13	5	3	ERG flicker: − 7.0/− 7.1; PHOT: − 10/− 8.3; SCOT: − 9.4/− 9.4	3	EPM2B: c.436G > A (p.Asp146Asn); c.838G > A (p.Glu280Lys)
P2	F	13	11	2	1	ERG flicker: − 2.8/− 3; PHOT: − 3.3/− 3.5; SCOT: − 4/− 3.9	0	EPM2A: c.323G > T (p.Arg108Leu)
P3	F	20	11	9	2	ERG flicker: − 5.8/− 6.9; PHOT: − 6.5/− 5.6; SCOT: − 4.8/− 4.3	3	EPM2A: c.323G > T (p.Arg108Leu)
P4	F	26	13	13	3	ERG flicker: − 3.9/− 2.9; PHOT: − 10/− 8.2; SCOT: − 7.4/− 9.5	4	EPM2A: c.243_246del (p.Asp82ArgfsTer7)
P5	F	20	11	9	2	ERG flicker: − 4.6/− 2.8; PHOT: − 4.5/− 5.6; SCOT: − 3.6/− 3.9	4	EPM2B: c.205C > G (p.Pro69Ala); c.826-829dup (p.Ala277AspfsTer23)
P6	M	20	16	4	3	ERG flicker: − 5.4/− 5.9; PHOT: − 7.6/− 9.4; SCOT: − 8.1/− 7.7	2	EPM2B: c.468_469del; (p.Pro69Ala) c.205C > G

*F* female, *ffERG* full-field electroretinogram, *FU* follow-up, *LE* left eye, *M* male, *PHOT* photopic, *Pt* patient, *RE* right eye, *SCOT*, scotopic, *y* years

**Table 2:** ffERG raw values observed in our cohort

Patient	Eye	DA 0.01 B-wave		DA 3.0 A-wave		DA 3.0 B-wave		Ficker Peak		LA 3.0 A-wave		LA 3.0 B-wave	
		ms	µV	ms	µV	ms	µV	ms	µV	ms	µV	ms	µV
*P2*	*L*	58.50	40.69	23.00	− 17.54	37.50	61.62	46.00	54.44	16.00	− 11.28	32.00	48.32
*P3*	*L*	67.00	36.37	18.00	− 12.37	41.50	25.96	45.50	26.15	16.50	− 4.54	34.00	19.49
*P1*	*L*	59.00	7.66	17.50	− 7.56	32.00	18.07	50.50	24.58	16.50	− 5.35	35.00	5.81
*P4*	*L*	84.00	13.38	28.50	− 15.91	45.50	36.23	54.00	21.02	14.00	− 2.10	36.50	6.84
*P5*	*L*	74.50	26.14	17.00	− 31.86	40.00	45.77	46.00	63.83	12.50	− 12.55	33.00	34.02
*P6*	*L*	59.00	13.47	17.00	− 15.04	35.00	26.33	45.50	18.08	13.50	− 3.78	33.50	13.90
Control	L	66.50	80.38	24.50	− 131.55	41.50	197.15	44.00	193.69	16.50	− 32.83	32.50	199.48

**Table 3:** Pearson’s correlation coefficients describing the relationship between cones and rods dysfunction and the disease stage

	*r*	*p*
Full-field ERG	0.597	0.211
ERG-LE	− 0.407	0.424
PHOT-LE	− 0.525	0.285
SCOT-LE	− 0.184	0.727

*ERG* electroretinogram, *LE* left eye, *PHOT* photopic, *RE* right eye, *SCOT* scotopic

**Table 4:** *T* tests results in the comparison between EPM2A and EPM2B groups on the type of mutation and the cones and rods dysfunction

	EPM2A group (mean ± SD)	EPM2B group (mean ± SD)	*p*
Full-field ERG	2.67 ± 0.58	2.00 ± 1.0	0.374
ERG-LE	− 5.67 ± 1.22	− 4.17 ± 1.52	0.253
PHOT-LE	− 7.37 ± 2.76	− 6.60 ± 3.35	0.775
SCOT-LE	− 7.03 ± 3.04	− 5.40 ± 1.78	0.467

*ERG* electroretinogram, *LE* left eye, *PHOT* photopic, *RE* right eye, *SCOT* scotopic

## Discussion

Both the retina and optic nerve share their embryological origin and vasculature with the brain, and the inner blood–retinal barrier and aqueous humour recall the blood–brain barrier and cerebrospinal fluid. For this reason, ophthalmological tests have been employed as a non- or minimally invasive tool for evaluating neural integrity in a wide range of neurological conditions which cause impairment in visual functions. Thus, an altered contrast sensitivity could be assessed in the early stages of Parkinson’s or Huntington’s diseases [20]. Moreover, OCT has recently been employed to identify specific markers of prediction, diagnosis, and progression of neurological conditions such as GLUT 1-deficiency.

Given that visual disturbances of LD patients are hardly evaluable through common ophthalmological evaluation, particularly in the early stages of the disease, to identify a safe, non-invasive, and rapid biological marker of LD we examined the retinal anatomy, FAF, SD-OCT, visual acuity, and colour visionof six patients with a genetically confirmed diagnosis of LD. During the ophthalmological evaluation, the retinal anatomy was unremarkable. FAF, SD-OCT (retinal lamination at the macula), visual acuity, and color vision was normal. In our cohort, we were able to perform the flashing light, because the patients had a mean disease stage of 2.67 and did minimally react to the flashing stimulus being sufficiently collaborative to perform an accurate ERG study. In a previous study by Korczyn and colleagues [21], it was reported a gradual improvement of the b-wave over time (from 30 s to 14 min); however, these b-waves still did not reach normal amplitudes. In our study, we performed standard ERGs following 20-min dark adaptation and observed reduction in both a- and b-wave amplitudes compared to controls who have ERG done identically. Furthermore, the intensities of light used for stimulation is different between the two studies.

**Fig. 4:**
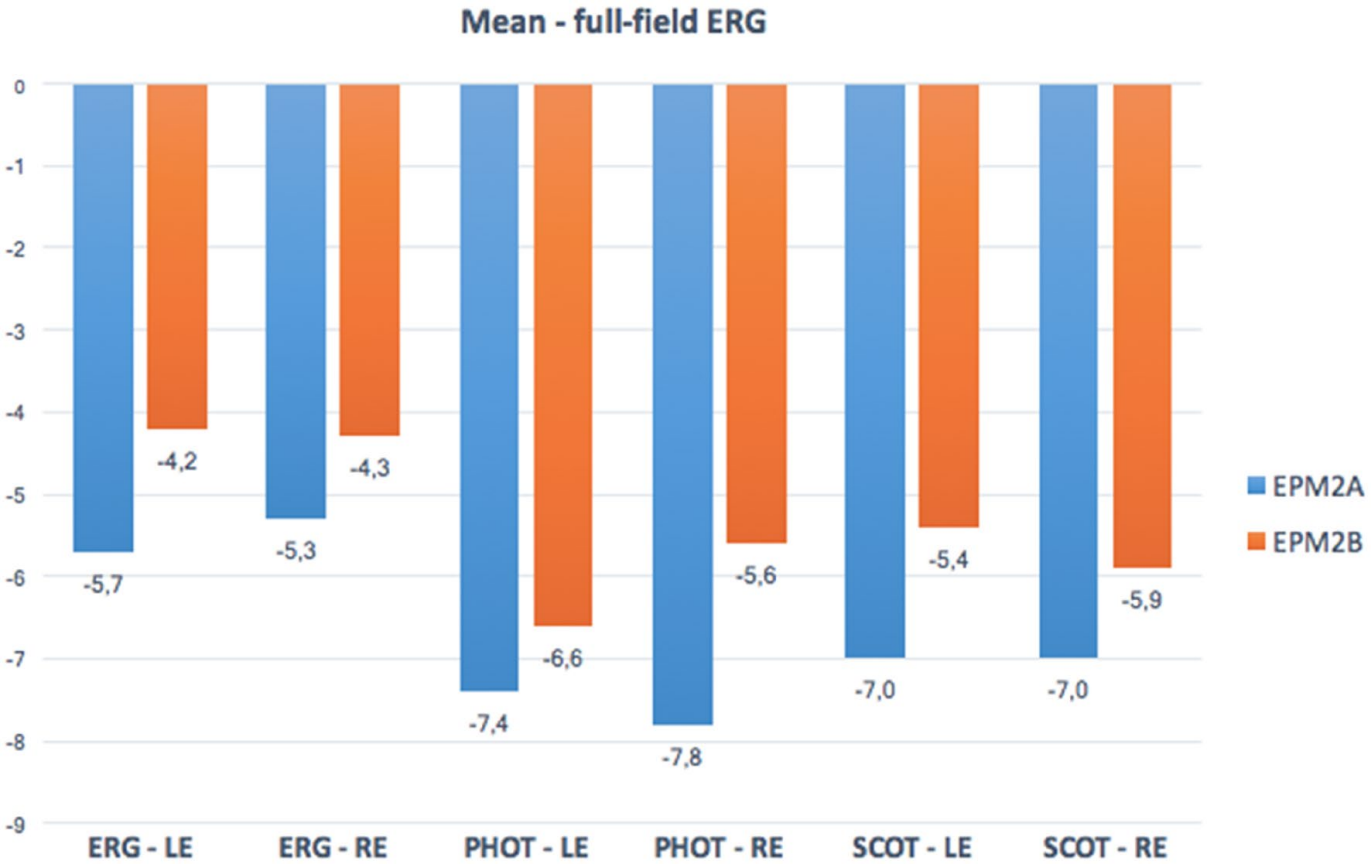
Mean full-field ERG results for the right and left eye each in the EPM2A and EPM2B subgroups. *LE* left eye, *RE* right eye, *ERG* electroretinogram, *PHOT* photopic, *SCOT* scotopic

**Fig. 5:**
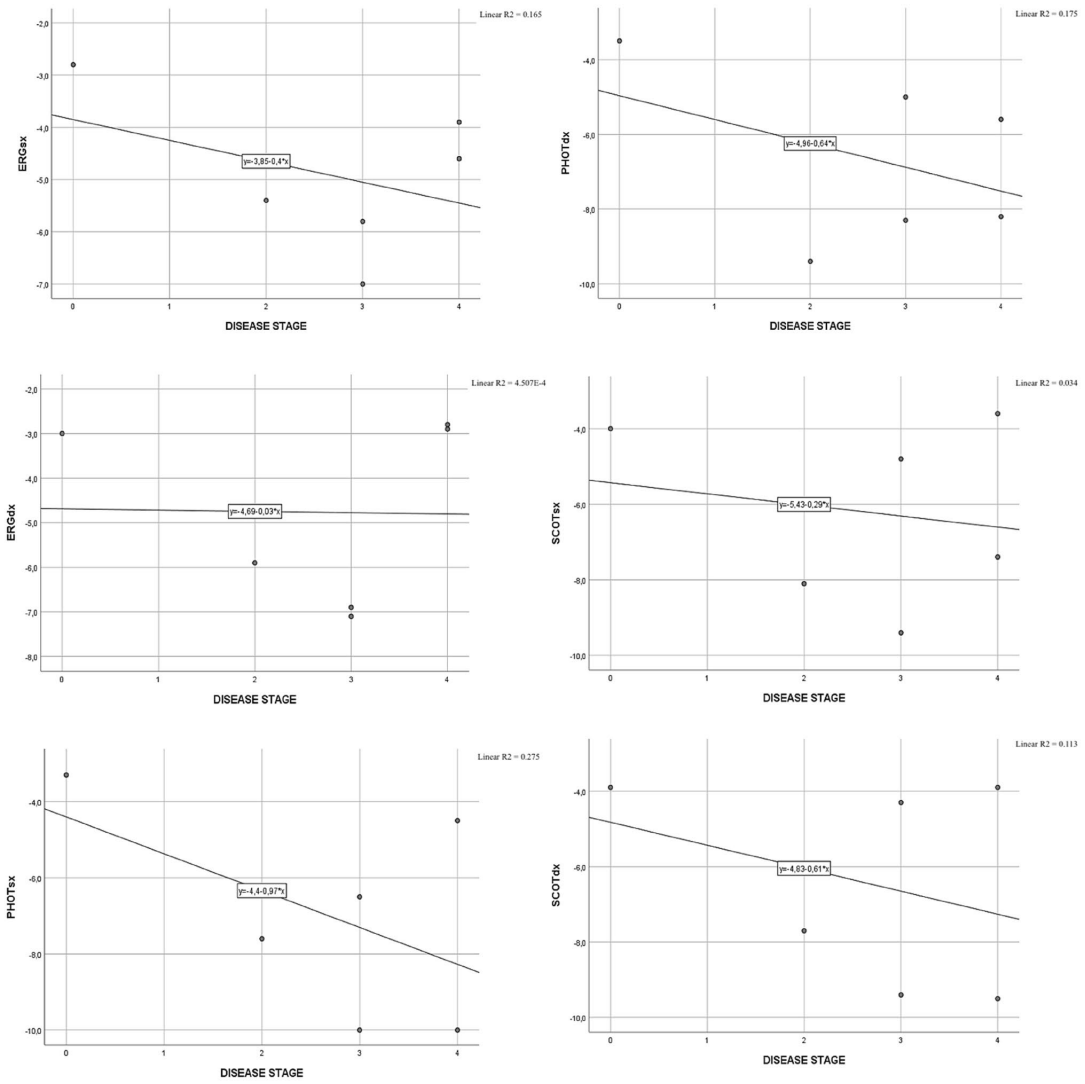
Graph showing the linear correlation between LD stage and the grade of photoceptors’ dysfunction for each eye for the different analyses. *LE* left eye, *RE* right eye, *ERG* electroretinogram, *PHOT* photopic, *SCOT* scotopic

The cones dysfunction was mainly in the moderate–severe stages (5/6 cases). Bipolar cell dysfunction observed at the ffERG may thus reflect the histological bipolar cells’ atrophy described in LD. Noteworthy, we identified a positive linear correlation between the disease stage and either the severity of cones dysfunction or the decreased rods photoreceptors a-wave amplitude function in the left eye (Figs. 2, 4, 5 ). Moreover, Fig. 1, shows how all ffERG traces are altered and reduced in amplitude in our patients as compared to healthy controls.

We were not able to perform Retinal Nerve Fiber Layer (RNFL) thickness measurements in patients due to invalidating psychomotor status of patients, but a recent case series showed reduced retinal thickness in two patients [22] and due to the small sample size, we failed to identify a statistical significance for the rod photoreceptors' a-wave amplitude function in the right eye. Nevertheless, we showed that *EPM2A* patients display a more severe dysfunction of both cones and rods photoreceptors (Figs. 1, 3) and show global cones and rods photoreceptors’ dysfunction in all the patients, confirming the preliminary results by Vincent et al. [19]. Although, these findings need to be evaluated more thoroughly in a large series of patients to ascertain whether they are consistent and, if yes if they progress longitudinally.

In summary, we bring further evidence of early retinal alterations in LD patients, regardless of the disease stage but being the dysfunction grade possibly related to disease duration. Hence, ffERG sets as an important tool to evaluate stages of LD, allowing to evaluate either natural or treatment-related disease progression in a minimally invasive way and to early intervene with gene-based therapies as soon as they will be affordable for patients.
